# Supplementary material for: Stochastic switching and analog-state programmable memristor and its utilization for homomorphic encryption hardware
Source: Nat Commun. 2024 Jul 26;15:6318. doi: 10.1038/s41467-024-50592-7 (PMC11282108; doi:10.1038/s41467-024-50592-7)
Supplement: Supplementary file 1 — Supplementary Information [file 41467_2024_50592_MOESM1_ESM.pdf]

Title:

Stochastic Switching and Analog-state Programmable Memristor and its Utilization for Homomorphic Encryption Hardware

Authors: *Woon Hyung Cheong*[1], *Jae Hyun In*[1], *Jae Bum Jeon*[1], *Geunyoung Kim*[1], *and Kyung Min Kim*[1,*]

1.
Department of Materials Science and Engineering
Korea Advanced Institute of Science and Technology (KAIST),
Daejeon 34141, Republic of Korea.

* Corresponding author

E-mail: km.kim@kaist.ac.kr

Supplementary Tables

Table S1. NIST randomness suit test results for evaluating random number generation performance required for homomorphic encryption. Each test for Mo-MCM was conducted under the condition of 1000 bits - 100 sequences

Test #	Test name	P-value (Mo-MCM)	Proportion (100 seq.)	Result (Mo-MCM)
**1**	Frequency (Monobit)	0.011412036	0.98	Pass
**2**	Frequency within a block	0.550717949	0.99	Pass
**3**	Runs	0.532759772	1.00	Pass
**4**	Longest run of ones in a block	0.348025369	0.98	Pass
**5**	Binary matrix rank	0.015710521	0.98	Pass
**6**	Discrete Fourier transform (Spectral)	0.771670504	0.99	Pass
**7**	Non-overlapping template matching	1.00	1.00	Pass
**8**	Maurer's universal	0.988785497	1.00	Pass
**9**	Linear complexity	0.999995031	1.00	Pass
**10**	Serial	0.761854261	0.99	Pass
**11**	Approximate entropy	0.999520079	0.99	Pass
**12**	Cumulative sums	0.017345555	0.97	Pass
**13**	Random excursions	0.950167133	0.98	Pass
**14**	Random excursions variant	0.910979293	0.99	Pass

Supplementary Figures

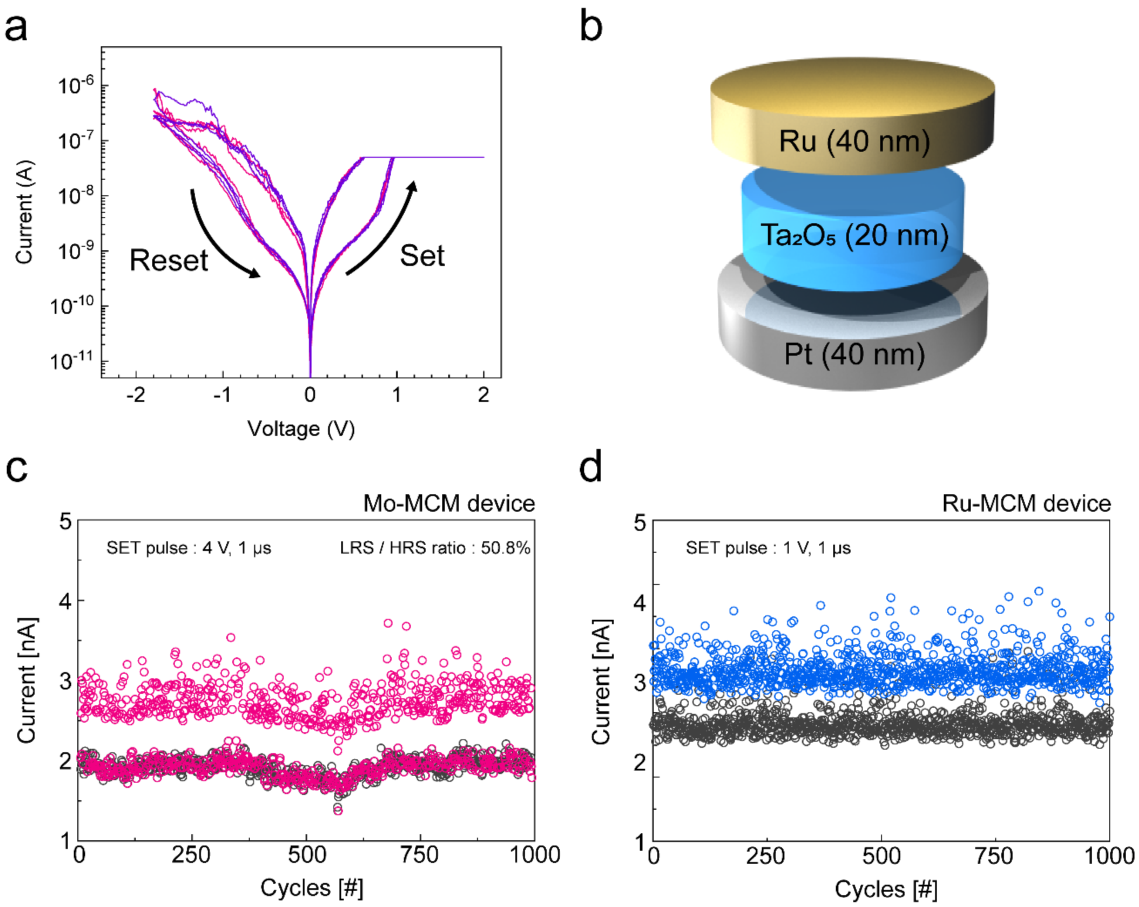

Fig. S1. Electrical characteristics of Ru-ECM (Pt/Ta_2O_5/Ru) memristors. a) I-V characteristic of a Ru-ECM memristor with 5 repetitions, which shows stable and repetitive switching behavior. b) Schematic image for a vertical structure of Ru-ECM memristors. The bottom Pt electrode was deposited using E-beam evaporation with a thickness of 40 nm, followed by a deposition of 20 nm of Ta_2O_5 using DC reactive sputtering with a Ta target. The top Ru electrode was deposited with a thickness of 40 nm using DC sputtering. c-d) Comparison results for stochastic switching characteristics of (c) Mo-MCM and (d) Ru-MCM. While Mo-MCM exhibited stochastic switching characteristics under a weak switching condition, Ru-MCM showed deterministic switching characteristics under a similar switching condition.

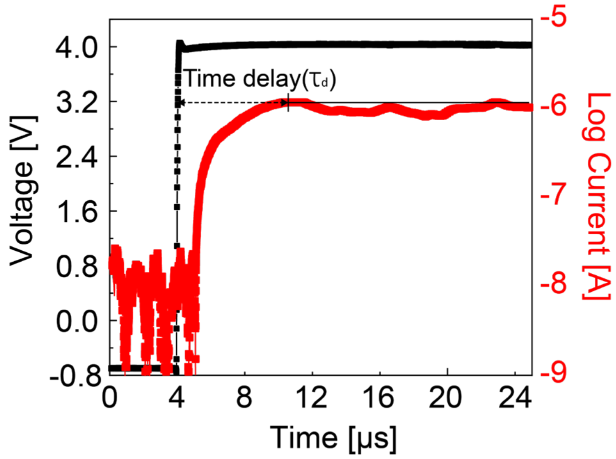

Fig. S2. A time-dependent current-voltage measurement for Ru-MCM. The τ_d of Ru-MCM was shorter than Mo-MCM, suggesting the higher W_0 in Ru-MCM.

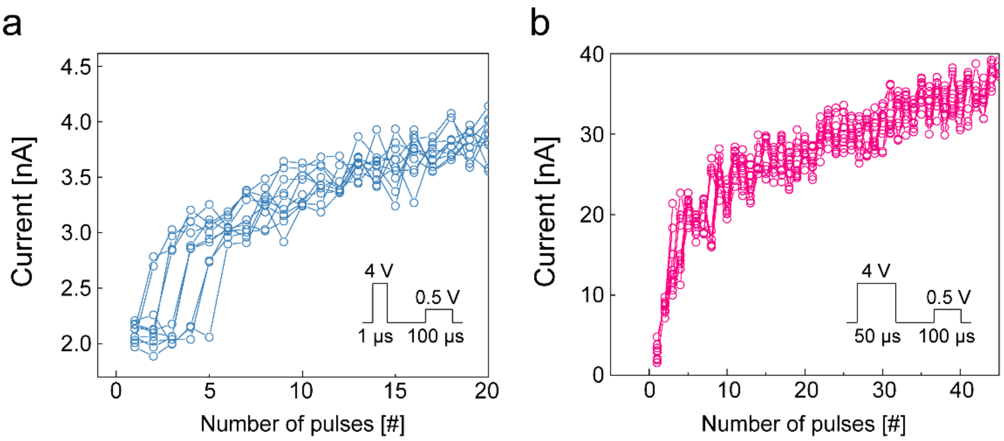

Fig. S3. The potentiation characteristics under 1 μs and 50 μs at 4 V. a) 10 cycles of potentiation characteristics for stochastic switching condition (4 V / 1 μs) and b) deterministic switching condition (4 V / 50 μs) are shown. there is variation during the initial switching, which is related to the probability of stochastic switching occurring. Once it has switched, the device exhibits typical potentiation behavior. In the deterministic switching condition, typical potentiation characteristics were observed. The saturation current at 20 pulses was about 4 nA in the stochastic switching condition and about 40 nA in the deterministic switching condition. This difference originated from the difference pulse widths for two conditions.

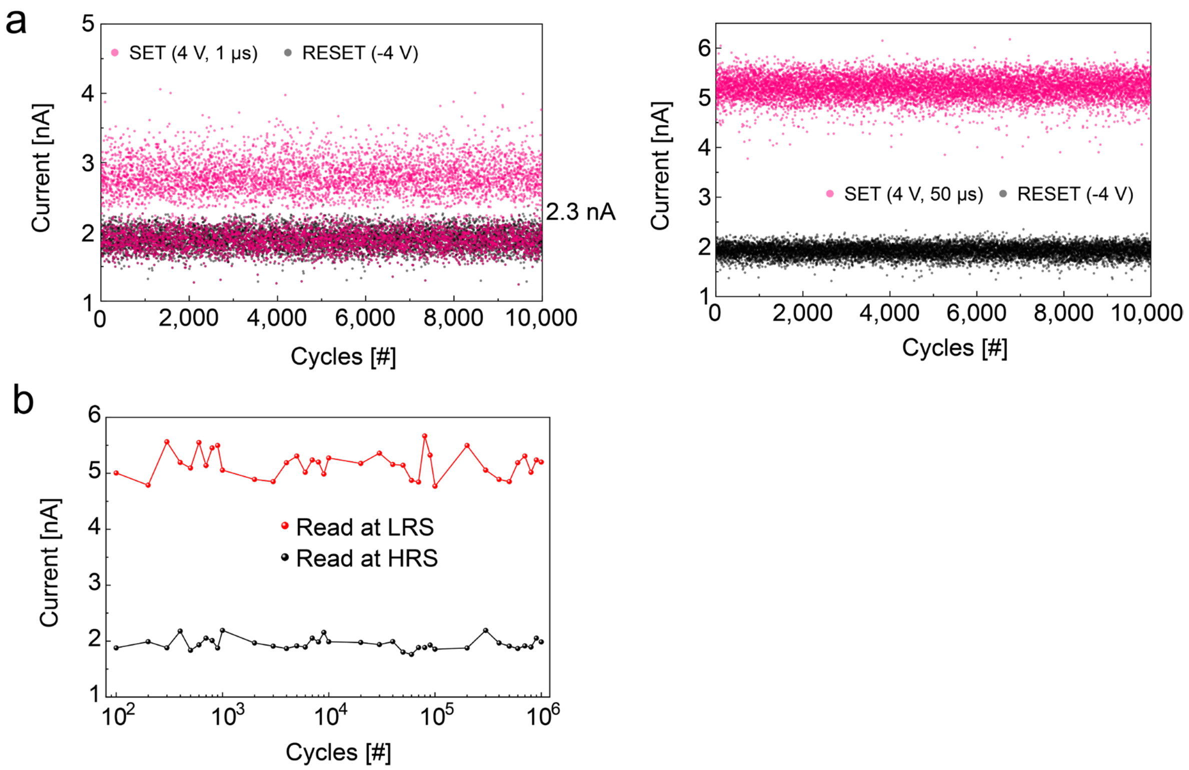

Fig. S4. Electrical endurance data of Mo-MCM. a) Endurance data for stochastic SET switching and analog computing with 10^4 cycles in a linear scale in two pulse schemes: (left) stochastic switching with 4V, ~ 1 µs pulses, (right) deterministic switching with 4 V, 50 µs pulses. Reset pulses were fixed to -4V, ~ 100 µs. b) Endurance of the deterministic switching in a log scale for 10^6 cycles.

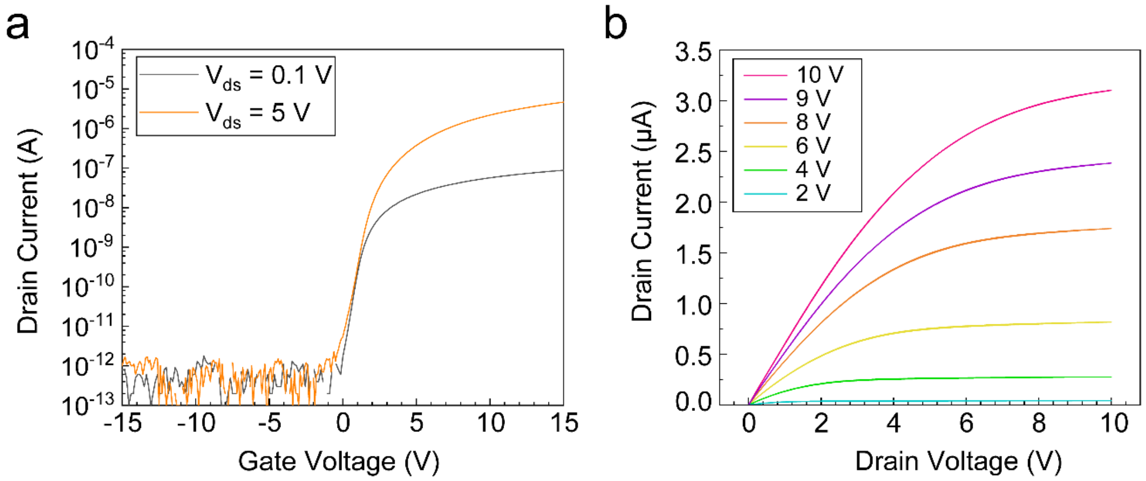

Fig. S5. Electrical characteristics of a-Si transistor. a) Transfer characteristics and b) Output characteristics of the a-Si transistor.

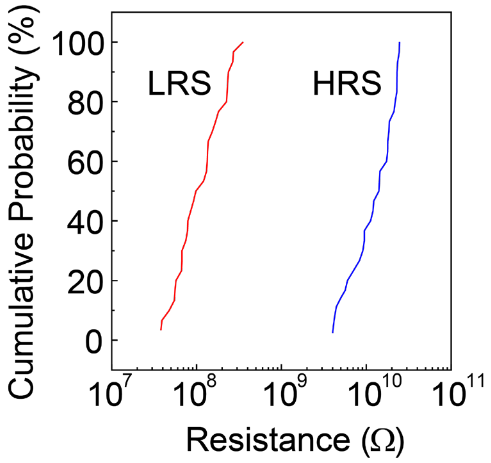

Fig. S6. Device uniformity for stable SET/RESET. Uniformity of programmed resistance states where a programming pulse condition of 4 V and 1 μs to an HRS is applied in 20 devices.

a

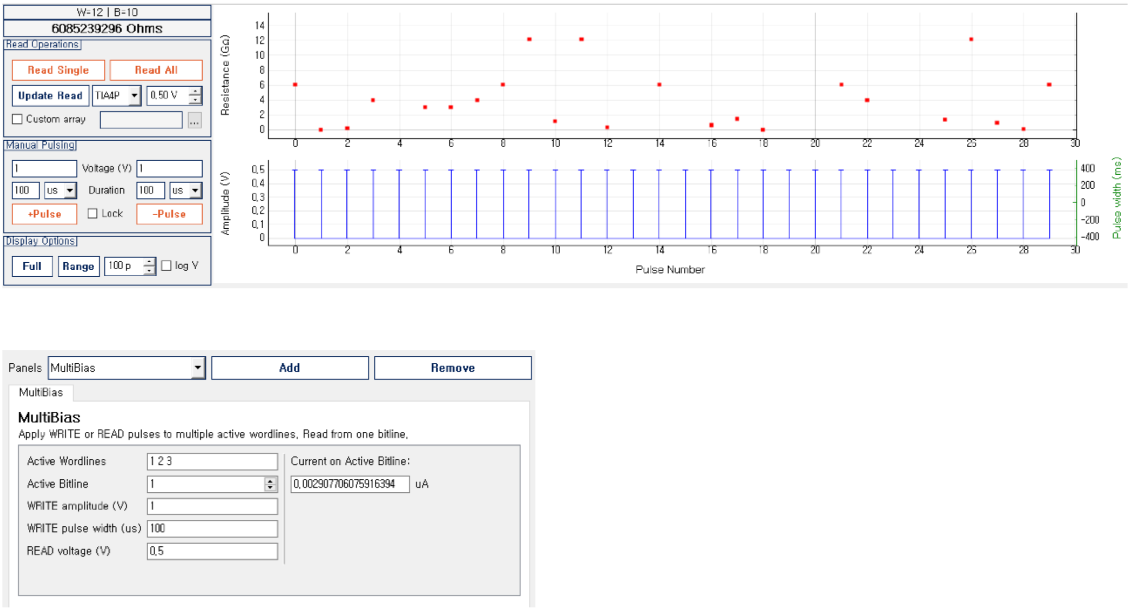

Fig. S7. Photograph of software interface for 3×3 1T1M array operation. a) Software schema when reading VMM (vector matrix operation) operation and programmed resistance values. b) When operating VMM, it is possible to choose and allocate wordline and bitline and set READ-WRITE voltage value.

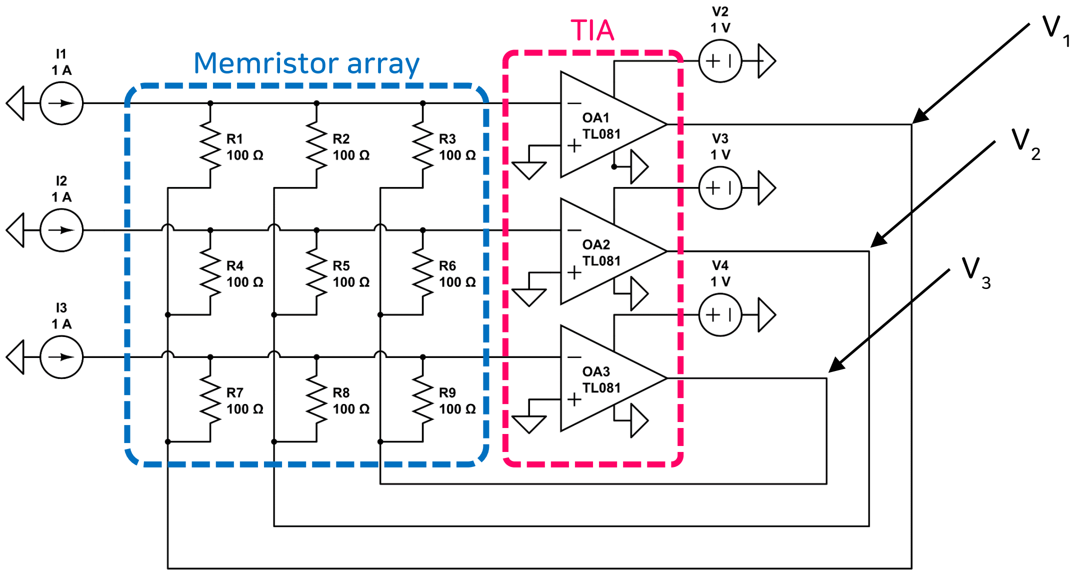

Fig. S8. Circuit for generating inverse matrix. The design of circuit is originated from Sun, Zhong et al. The value of the inverse matrix of the matrix programmed in the memristor array appears as the voltage value ($V_{1\text{-}3}$) output through the transimpedance amplifier. For the inverse matrix operation, the current values ($I_{1\text{-}3}$) corresponding to the columns of the unit matrix (E) are sequentially entered into each row. Verification of this circuit was performed using linear technology simulation program with integrated circuit emphasis (LTSPICE) memristor library.
